# Conducting rigorous research with subgroups of at-risk youth: lessons learned from a teen pregnancy prevention project in Alaska

**DOI:** 10.3402/ijch.v75.31776

**Published:** 2016-12-09

**Authors:** Kathryn Hohman-Billmeier, Margaret Nye, Stephanie Martin

**Affiliations:** Institute of Social and Economic Research, University of Alaska Anchorage, Anchorage, AK, USA

**Keywords:** at-risk youth, randomized control trials, Alaska youth, peer education, evidence based interventions, sexual health education curriculum

## Abstract

In 2010, Alaska Department of Health and Social Services (DHSS) received federal funding to test an evidence-based teen pregnancy prevention program. The grant required a major modification to an existing program and a randomized control trial (RCT) to test its effectiveness. As the major modifications, Alaska used peer educators instead of adults to deliver the program to youth aged 14–19 instead of the original curriculum intended age range of 12–14. Cultural and approach adaptations were included as well. After 4 years of implementation and data collection, the sample was too small to provide statistically significant results. The lack of findings gave no information about the modification, nor any explanation of how the curriculum was received, or reasons for the small sample. This paper reports on a case study follow-up to the RCT to better understand outcome and implementation results. For this study, researchers reviewed project documents and interviewed peer educators, state and local staff, and evaluators. Three themes emerged from the data: (a) the professional growth of peer educators and development of peer education, (b) difficulties resulting from curriculum content, especially for subpopulations of sexually active youth, youth identified as lesbian, gay, bisexual, transgender, queer, intersex and/or asexual, pregnant, and parenting youth and (c) the appropriateness of an RCT with subpopulations of at-risk youth. Three recommendations emerged from the case study. First, including as many stakeholders as possible in the program and evaluation design phases is essential, and must be supported by appropriate funding streams and training. Second, there must be recognition of the multiple small subpopulations found in Alaska when adapting programs designed for a larger and more homogeneous population. Third, RCTs may not be appropriate for all population subgroups.

In 2010, Alaska Department of Health and Social Services (DHSS), Adolescent Health Program in the Division of Public Health, Section of Women’s, Children’s and Family Health, was awarded funding to implement an evidence-based program to reduce teen pregnancy and sexually transmitted infection (STI) rates. The funding was part of the teen pregnancy prevention (TPP) initiative within US Department of Health and Human Services (HHS), Office of Adolescent Health (OAH). The purpose of the funding was to expand the use of evidence-based programming in TPP. To be considered evidence-based, programs undergo a rigorous evaluation, usually a randomized control trial (RCT). Prior to the initiative, most of the TPP programs considered to be evidence-based had been implemented and evaluated only by their developers, usually with one population (e.g. African-Americans, aged 12–14) and in one setting (e.g. urban middle schools (17,18,19,20)). Under the award, grant recipients tested program effectiveness with different populations, in different settings, and tested whether programs could be implemented with fidelity. Each award also required that a key innovation be incorporated into the program. To meet evidence standards, awards also required rigorous evaluations of behavioural outcomes and a thorough program implementation evaluation.

The Adolescent Health Program saw this as an opportunity to address Alaska’s combination of high teen pregnancy, STI rates and lack of access to information. The birth rate for teens 15–19 in Alaska was 42.7 per 1,000 in 2008 (prior to the start of the program) (1). At that time, within the state, rates were as high as 109.2 per 1,000 for Alaska Native youth in some rural areas (1). These compare to 40.2 per 1,000 for the US (2). Alaska ranked first or second in the country in chlamydia rates from 2000 to 2010 (3). Alaska also has minimal health education standards, requiring only one credit for health or physical education as a high school graduation requirement (Alaska State Statute 4 AAC 06.075).

Initially, the Adolescent Health Program selected Making Proud Choices! (MPC), a safer sex program, from the list of approved evidence-based curricula (4). The innovation was to use peers instead of adults as teachers. A year into the grant, in 2011, DHSS Administration required an abstinence-primary curriculum or a return of the funding to OAH. As a result, Select Media, the developers of MPC, recommended “Promoting Health Among Teens: Comprehensive Abstinence and Safer Sex Intervention” (PHAT). PHAT is a combination of MPC, a safer sex program, and PHAT, an abstinence-only program.^1^ The curriculum was approved provided that PHAT implementations were not to be conducted in traditional schools or with participants younger than 14. The approval also required that peer educators use their fingers instead of a penis model to demonstrate how to use a condom. In addition to the delay while selecting a new program, these restrictions limited our study population.

An additional limiting factor was the discovery, after implementation had begun, that the study design with no intervention for the control group could not be approved for use with incarcerated youth due to federal HHS regulations on the protection of research subjects. Grantees had planned for substantial participation by incarcerated youth when they estimated how many youth they would be able to recruit.^2^ In partnership with the Alaska grant recipients (“grantees”) Alaska Youth Advocates in Anchorage, Kachemak Bay Family Planning Clinic in Homer, and Tundra Women’s Coalition in Bethel, DHSS adapted PHAT to include peer-initiated and strengths-based language (like “us” and “we”), cultural practices (like the use of a talking stick and reduction in metaphor use) and LGBTQAI sensitive statements (like the use of the word “partner” instead of “boyfriend” or “girlfriend”). Once the adaptions were complete, the curriculum was renamed Alaska Promoting Health Among Teens! (AKPHAT). An additional grant recipient, Cook Inlet Tribal Council, Inc., was awarded funds in 2012.

From 2011 to 2015, four non-profit grantees worked in partnerships with local community agencies to pilot and fully implement AKPHAT. Peer educators were local youth, aged 16–21, and were true peers (i.e. at-risk and/or homeless youth). The program was delivered after school, during weekends and during holiday and summer breaks, and in the case of one alternative high school, during school. The outcome evaluation for AKPHAT was an RCT with individual level assignment (into treatment or control groups).

The end of the RCT marks the start of this case study. The RCT had unexpectedly low recruitment and high attrition rates, leaving too little statistical power to detect any effect. We wanted to know more about peer educators, to understand what our study population thought about how the curriculum worked, and to understand why so few people participated.

## Methods

This study extends the work of others (5–8) incorporating mixed methods into RCTs in order to improve evidence-based research. Using a case study (9) approach with qualitative descriptive methods (10,11) allowed this research to look at the experiences of people directly involved in the development and implementation of AKPHAT. The study team employed a multimodal approach to conduct semi-structured interviews including, but not limited to, face-to-face, telephonic, social media, and email correspondence. Interviewers worked from a set of open-ended questions meant to foster a broader conversation about the factors that contributed to the low participation rate in the AKPHAT program, and the resulting inability to detect an effect of participation on sexual behaviour. Key informants were identified by their participation in the program and/or its evaluation, and included grantee program supervisory staff, peer educators, community partners, Institute of Social and Economic Research evaluation staff, DHSS staff and any additional project personnel involved in program design and/or implementation. The study also includes information from program documents including proposals, contracts, correspondence, meeting minutes, memoranda of agreement, reports and fidelity monitoring data. Face-to-face interviews were in neutral locations minimizing the possibility of influence from other program participants and/or support staff. Table I shows the number of interviewees by their role in the program.

**Table I:** Interviewees and their role in AKPHAT

	Manager/supervisor	Peer educator	Community partner
Grantees	10	13	7
State of Alaska	3		
Evaluation	3		

Compared to survey format, semi-structured interviews provide more detailed information. Open-ended questions allow respondents a broader range for making sense of their own experiences. Additionally, semi-structured interview designs allow respondents to incorporate related topics and ideas not in the interview script. Researchers started from six broad themes: implementation, recruiting, communication, RCT and evaluation, training and curriculum. Two additional themes emerged from the interviews: growth of the grantee organizations and the difficulties of managing the process of a complex 5-year grant. From these eight themes, multiple subthemes were identified. Researchers narrowed the discussions to the themes most often discussed across the categories of participants.

In November and December 2014, researchers conducted 36 semi-structured interviews. Each was recorded, transcribed, entered into Atlas.ti and coded according to themes. Researchers analysed data across several categories: the four grantee organizations, grantee supervisors and directors, peer educators, community partners, rural and urban settings, and primary Alaska Native youth serving organizations or organizations serving all youth.

### Subgroups of at-risk youth

In our review of the literature, we have not found other uses of the phrase “subgroups of at-risk youth.” Damianakis and Woodford (12) use the phrase “small connected communities” to discuss how to uphold ethical issues such as protecting confidentiality when conducting qualitative research with community members who have relationships with one another. Other scholars discuss research among “small and close knit Native communities” (13) and “geographically bounded and tightly knit” communities (14), though those definitions do not necessarily capture the essence of the populations within which the AKPHAT program was implemented. “Subgroups of at-risk youth,” instead, offers a framework for discussing the various and unique groups of youth that participated in the AKPHAT program in Alaska. Between 2011 and 2015, grantees implementing the AKPHAT program targeted at-risk, vulnerable and underserved youth populations. This included Alaska Native youth, youth in foster care, and homeless and transient youth in both urban and rural Alaska. Additionally, lesbian, gay, bisexual, transgender, queer, intersex and asexual (LGBTQIA) youth, pregnant women or mothers under 21 years of age, and youth residing in areas with high teen birth rates were participants in the program. Working to implement a universal curriculum with each of these subgroups presented a unique set of challenges for grantees, peer educators and community partners.

## Results

Grantees shared mixed responses to questions about the AKPHAT program. Across settings and clients, the most widely discussed topics were curriculum relevance and peer educator growth.

For grantees working primarily in rural Alaska, the top three themes included curriculum relevance, curriculum improvements and the judgmental tone of the curriculum. This reflects more broadly on the long-standing tension between Alaska Native ways of knowing and being and one-size-fits-all program interventions. Peer educators working in rural communities and/or with Alaska Native youth organizations reported that AKPHAT curriculum could come across as judgmental and irrelevant, or shaming; however, they also reported making modifications that included culturally appropriate ways of delivering the information that, in their opinion, impacted positively the way that it was received by participants. Examples of such modifications included adding more inclusive language (i.e. adding LGBTQIA to the curriculum), changing judgmental language around pregnancy when teen mothers were participants, and including Yup’ik words and phrases for participants whose first language is not English.

Supervisors and directors reflected on the project’s wider scope, peer educator success, length of time required for the institutional review board (IRB) and tribal review processes, peer educator growth and initial proposal development. By comparison, peer educators across all sites discussed their professional growth, curriculum relevance, training by their grantee, participant growth and improvements made to the curriculum.

Interviews with state employees revealed a focus on launching AKPHAT and the political environment in which it was implemented. Community partners discussed difficulties recruiting busy teens, logistical challenges of working with a transient population, overall teen participant recruitment, difficulties recruiting and implementing an RCT and the associated lack of an activity for the control group in the RCT.

### Peer educator growth and success

Peer educator curriculum delivery was an important program adaptation. Grantees were encouraged to use positive youth development principles to mentor and support peer educators. Across all categories of interview respondents, peer education was identified as an overall success of the program. Peer educators were described by others, and described themselves, as confident, knowledgeable and mature youth in their communities. Site supervisors and community partners reflected on how these youths translated their skills beyond AKPHAT into other aspects of the community, generating positive community development. Supervisors reflected:

As far as the peer educators, I feel like they have to step up in so many ways and you just see them transformed … it was just really wonderful to see how they integrated it into their lives. And now they use it to reach out to people and situations and things that did not have anything to do with sexual health but because they had this position where they were a peer educator, they took that farther in so many other parts of their lives and really I think helped people, helped their peers in a lot of different ways.

Well, it’s hard to find jobs out here for teens and it’s hard to find a job that’s like going to actually give you skills other than like working registers, so that’s been … just a really good opportunity for some of our youth to have … a really compelling job. Public speaking skills, confidence, peer leadership. … I’m really grateful that we’ve been able to give them this opportunity.

Peer educators described their experiences: “I have boosted my confidence and my ability to communicate … and I am confident in what I know … I know all this information now about sexual health and I feel like I’m able to make better choices about sexual health because of it.” Some noted how their experience provided them with a broader perspective on their lives. According to one, “I’m learning how to be a positive influence in the community and that makes an extremely big impact as you go onto to more typically adult positions.”

### Curriculum

Fidelity monitoring and an implementation evaluation conducted prior to this case study demonstrated that grantees implemented AKPHAT according to design. However, in this case study, interviewees revealed issues with the curriculum that show difficulties meeting the needs of subgroups. Categories discussed included curriculum relevance, curriculum improvement and the curriculum as judgmental.

The original modification of using a talking stick did not arise in the interviews. The views expressed about cultural relevance and use of judgmental language conveyed the original curriculum modifications were not enough.

Discussing the curriculum directly, a peer educator summarizes her perspective on its delivery in Alaska: “I’m not saying the curriculum is bad, but … the curriculum doesn’t work for every place and every person every time.” Another peer educator noted the exclusion of particular subgroups with whom the grantee worked, “It just seems like we could have given more information about LGBTQIA people without the negative context that may come with it.”

Furthermore, restricting program participation to youth aged 14 and older meant that the study population was more likely to be sexually active (15), thus negating the message of the abstinence-primary curriculum. As one peer educator reflected,

I felt like people who decided to practice sex or safe sex were a little put down in the curriculum – like always saying that responsible people choose to practice abstinence … When in reality, I think everyone should be allowed to be sexual beings and be who they are.

Other grantees suggested changes to the curriculum language, “taking out a lot of shame and blame language … that’s not what we’re there to do. We’re there to share evidence-based information.”

The curriculum states: “this partially scripted role-play activity provides an opportunity for participants to be advocates for abstinence and to further internalize this option as the healthiest choice for people their age” (16).

This activity stands in contrast with culture and practice in parts of Alaska, particularly among the age group and subpopulation to which the curriculum was delivered. One peer educator discusses the implications of stressing abstinence-primary messaging with subgroups of sexually active and parenting youth:

I definitely did not like a lot of things in the curriculum. A lot of things from the curriculum and our culture did not match up. Like in our culture … children are very, very much valued and it’s a norm, you know, to have a baby early or “young” and then in the curriculum I kind of felt like it was shaming those people or in a way making them feel bad for the decisions that they made.

Peer educators became more comfortable with the curriculum as they modified language around teen pregnancy, LGBTQIA inclusion, and safe sex. Additionally, in Yup’ik-speaking regions, peer educators reported that the use of Yup’ik words and phrases increased the retention of information for participants whose first language is not English.

Other interviewees further discussed the relevance of the curriculum. One site supervisor reflected on the appropriateness of the curriculum, as delivered with fidelity, for youth in rural Alaska:

I don’t even know that PHAT curriculum as it exists right now works out here. It’s just so culturally alien. Like the videos … they’re [of] inner city youth … if you want to teach a safe sex curriculum in rural Alaska to [Alaska] Native children, then it should be (Alaska) Native youth videos that they’re seeing.

In addition to reflections on the curriculum itself, interviewees commented on the restrictions imposed on the AKPHAT program due to the political climate and the effects that those changes had on implementing in their communities. Peer educators and site supervisors discussed the lack of a penis model during AKPHAT condom demonstration modules. That peer educators were unable to demonstrate the proper way to use a condom impacts the delivery of medically accurate information. Regarding the restrictions, one peer educator lamented,

I think the lack of a condom model is silly … Using two fingers … something is missing and what’s missing is a penis … When you start to roll a condom down two fingers, it doesn’t feel like anything’s happened … but when you roll a condom down a model penis … then you’re ready for sex, because now there’s a condom on a penis and that’s how it’s supposed to be.

Finally, interviewees discussed the difficulties of implementing the 12-hour curriculum. Not only were age limits of participants increased,^3^ some effects of which were discussed above, but also peer educators were unable to deliver curriculum in traditional school settings. Site supervisors and peer educators voiced that asking youth to volunteer 12 hours of their time to sex education after school, on the weekends, or over holiday or summer break, was a challenge. Interviewees cited jobs, subsistence hunting and fishing activities, and after-school sports and school-related activities as competitors for the 12-hour curriculum implementation. This, in turn, impacted the number of youth that grantees were able to reach.

### RCT design

With funding, grantees agreed to participate in the RCT. Early in the process, program implementers, grantees and the evaluation team decided to not provide an alternate curriculum or activity for the control group because of staffing, logistics and funding difficulties.

Some grantees reported that the RCT made it difficult to recruit both community partners and participants. One site supervisor noted that community partners were hesitant because they wanted all the youth in their community to be educated, not just some of them. In some instances, community partners expressed interest, but only wanted to implement in the last year after randomization ended. One interviewee reflected,

We’re creating some haves and have-nots in terms of what we’re offering for service … in research [this] is normal, but when it actually comes to people … especially for a social service organization, [is] not so easy to swallow.

Another site supervisor shared that some community partners felt that offering one group of youth an educational experience without offering the other (control) group a comparable benefit was “irresponsible” of the grantee. Grantees reflected that they were not only asking community partners to set aside their hesitation about a sensitive topic (sex) in order to educate youth, but that they were having to also explain the randomization process and convince them to participate in it. Grantees working to recruit community partners shared that they were often in the difficult position of having to explain and defend research design choices that were not always clear or viewed as equitable or ethical to potential community partners.

Other interviewees discussed challenges in recruiting program participants. Peer educators and site supervisors indicated that many youth expressed interest in the AKPHAT program as couples or friends, and wanted to take the class together. However, in some cases, youth either did not sign up out of fear of being randomized out of the same group as their friend/significant other, or dropped out after they were randomized into different groups. One peer educator shared,

… if you recruit like two buddies or a couple and you have to tell them “well, one of you might make it into the control group, so you guys might be split-up” and that has disappointed some people. I think it’s turned some people off, too, from taking the class.

Other interviewees reflected on the impact that randomization had on their at-risk youth. As these two community partners describe, being randomized into the control group led to, in their opinion, a re-traumatization for their youth population, especially when RCT was not well explained to them:

… for those youth that came and then weren’t [randomized into] a part of the [treatment] group … people were crying and really upset. They felt like they were rejected …

… for some of them they have really tender hearts and egos … [for] my alternative at-risk kids, that’s hard to take.

Finally, RCT designs have largely not represented participatory, collaborative research. Alaska Native populations have, historically, been the objects of study rather than collaborators in research design. In case study interviews, some Alaska Native organizations expressed discomfort with splitting groups into treatment and control, where the control group receives no intervention. When asked about participation in this RCT, one community partner expressed frustration about the research process:

… especially in this area, there’s been so many studies done … in the past. It’s like an institutional racism thing, like people are studied here all the time and they’re given programs all the time. With working with some of the agencies, it’s like “really? You want to do another study?” That just creates ire. Like “study somebody else” is that kind of attitude. So I would avoid doing a study like this in the future.

As this interviewee makes clear, understanding historical context and respecting individuals as collaborators in projects that seek social change in their own communities cannot be ignored. Issues of beneficence and justice must be addressed.

## Discussion

The quantitative analysis was unable to determine whether peer educators were effective as teachers. However, this research shows that use of peer educators appears to be one way to effectively reach subgroups within at-risk groups. Peer educators in AKPHAT were true peers of participants; therefore, peer educators also benefitted. They were able to access meaningful opportunities, services and resources; practice developing professional relationships with their peers and adults as well as with themselves; and adopt quality leadership skills and act as ambassadors and peer counsellors to other youth in their communities.

The grant allowed for curriculum selection from a list of evidence-based programs. The selection was narrowed one year into the grant by a requirement to use an abstinence-primary curriculum. Case study findings strongly suggest the curriculum was not the best fit for the target populations. According to interviews, parts of the curriculum were not appropriate or useful for subgroups. The curriculum did not include LGBTQIA youth, pregnant or parenting youth. It attached concepts of “proud” and “responsible” to abstinence in a group where many were sexually active, and of those, not everyone by choice. The curriculum may be better suited to a younger age group. However, these are issues for evidence-based programs, most of which are prepackaged and required to be delivered as they were designed. Working with communities to develop and pilot effective culturally-appropriate, age-appropriate and targeted population-appropriate factors into adaptations prior to implementation was theoretically possible, but the time line and funding constraints of the federal grant program precluded taking that approach. This would be an important consideration in funding programs going forward.

The RCT evaluation, the traditional gold standard for rigorous evaluation, was the biggest barrier to participation. The RCT process impacted AKPHAT effectiveness, in particular, recruiting community partners and participants. The fact that an activity was not made available for the control group amounted to a denial of services; small communities and Alaska Native communities did not want to “split” their populations so instead opted out of the AKPHAT program altogether. Similarly, teens did not want to sign up for a program where they might be separated from their friends through the randomization process. Co-development of the research design with participating communities might have identified effective control group interventions, and found other ways to make the RCT more acceptable; but as above with cultural adaptations, such an approach was not feasible in the federal grant that funded this program.

Future studies could greatly benefit from including communities in the selection, and further modification, of evidence-based programs to be implemented. They could also benefit from including communities in evaluation planning and design. In so doing, communities where evidence-based programs are being implemented could provide critical input for designing appropriate, and inclusive, RCT evaluations among their populations. Other options could include the use of a randomized cluster design at the community level, quasi-experimental designs or indigenous research methodologies, particularly in Alaska Native communities.

Ultimately, quantitative analysis of AKPHAT suggests that the program was unsuccessful as a result of low participation and the failure to demonstrate effect. However, the power of the case study allowed for us to explore more deeply meanings of successes and failures of the AKPHAT program among those who were direct participants in the program (i.e. grantees, peer educators and community partners). Interviews generated from the case study indicate that the peer educator model has been a success in the communities where AKPHAT was implemented. Conversely, the combination of an individualized RCT model and the curriculum’s relevance among Alaska Native youth and youth aged 14 and older impacted the program’s ability to attract, retain and resonate with those subgroups of at-risk youth. Finally, rigorous research studies utilizing an individualized randomized control treatment method must target a larger pool of at-risk youth in order to demonstrate a wider impact.

Through the case study, the following lessons were learnt:

- Community involvement and input in choosing a curriculum and evaluation design is essential.
- Implementation timelines must include sufficient time for IRB and tribal approvals which are time-consuming.
- Evidence-based curricula need to be adapted to be culturally appropriate and take into consideration the target populations’ culture, age and risk factors.
- Other research designs should be considered, but if the RCT design is chosen, control groups should receive an alternate intervention.
- For the purpose of this OAH grant, one size does not fit all; just because a curriculum is evidence-based in one population does not mean that there will be evidence of effectiveness in another population.

## Footnotes

1 AKPHAT contains 12 modules, each designed to last 1 hour. According to developers, it can be administered over a few days or several weeks.

2 The original target was to implement with 1,008 participants per year (252 total participants per grantee at four grantee sites), but participants only totaled 302 with 155 and 124 three- and six-month follow-up surveys.

3 From the original target audience of 13–19 to ages 14–19 instead.
